# A proposal for a national registry on chronic migraines

**DOI:** 10.1186/1129-2377-16-S1-A40

**Published:** 2015-09-28

**Authors:** Nicola Vanacore, Sabina Cevoli, Paola Torelli, Cinzia Aurilia, Gabriella Egeo, Luisa Fofi, Licia Grazzi, Gennaro Bussone, Gian Camillo Manzoni, Pietro Cortelli, Piero Barbanti

**Affiliations:** National Centre of Epidemiology, National Institute of Health, Rome, Italy; Department of Neurological Sciences, University of Bologna Medical School, Bologna, Italy; Department of Clinical and Experimental Medicine, Headache Centre, University of Parma, Parma, Italy; Department of Neurological Motor and Sensorial Sciences, IRCCS San Raffaele Pisana, Rome, Italy; C. Besta Neurological Institute and Foundation, Milan, Italy

According to the existing classification chronic migraine (CM) is a primary headache that occurs on 15 or more days per month for more than 3 months and has the features of migraine on at least 8 days per month. In Europe, CM prevalence ranges from 2.7% to 4.7%. CM is an extremely disabling disorder associated with significant functional impairment. The 2010 Global Burden of Disease Survey conducted by the WHO listed migraine as the 7th cause of disability in the world, responsible for 2.9% of all years of life lost to disability. Unfortunately, however, in its current definition CM includes subgroups of patients with very different levels of severity and outcome. Failing to recognize them entails highly negative repercussions on research and clinical practice, with inadequate patient management and lack of cost-effectiveness. Therefore, it is essential that these subgroups of subjects be clearly identified to optimize clinical management, rationalize the allocation of economic resources, provide specific clinical and health care procedures, and clarify pathogenetic mechanisms. The clinical registry will include the data of all patients with CM, aged 18 or over, seen at the Headache Centres of Parma, Bologna, Rome, and Milan. The diagnosis of CM will be made by a headache specialist based on the diagnostic criteria of the ICHD-III beta (2013) and on the CM classification proposed by Manzoni et al[1]. The principal aim of the clinical registry will be to identify still undefined subgroups of subjects with CM through a specially designed clinical registry to be applied in a large number of outpatients. The registry will make it possible to collect: personal and social patient data, as well as data about their physiological conditions and non-essential habits; clinical features of headache (before and after its evolution to chronicity); any factors concomitant with the headache's evolution to chronicity (i.e., medication overuse, life events, hypertension); comorbidity and injuries; headache-related disability (MIDAS, WHO-DAS-II, MSQ); quality of life (SF36); symptomatic and preventive treatments; medication overuse; visits and hospitalizations for CM; and recognized sickness and invalidity allowances. The creation of a large-scale registry will make it possible to identify specific CM (i.e., refractory CM) subgroups, based on clinical and biological features. It will also make it possible to identify areas with lack of or inadequate health care provision and waste of resources (i.e., useless examinations or treatments), eventually helping to improve CM management and ensure health care procedures that are more appropriate.

## References

[1] Manzoni GC, Bonavita V, Bussone G, Cortelli P, Narbone MC, Cevoli S, D'Amico D, De Simone R, Torelli P, ANIRCEF (Associazione Neurologica Italiana Ricerca Cefalee) (2011). Chronic migraine classification: current knowledge and future perspectives. J Headache Pain.

